# Diversity of *Cytauxzoon* spp. (Piroplasmida: Theileriidae) in Wild Felids from Brazil and Argentina

**DOI:** 10.3390/pathogens14020148

**Published:** 2025-02-04

**Authors:** Ana Cláudia Calchi, Joares A. May-Júnior, Vinícius Baggio-Souza, Laura Berger, Renata Fagundes-Moreira, Rafaela Mallmann-Bohn, Laíza de Queiroz Viana Braga, Murillo Daparé Kirnew, Matheus Folgearini Silveira, Roberto Andres Navarrete Ampuero, Charlotte O. Moore, Ricardo Bassini-Silva, Heitor Miraglia Herrera, Edward Bealmear Breitschwerdt, Ricardo G. Maggi, Eduardo Eizirik, Rosangela Zacarias Machado, Fabiana Lopes Rocha, João Fabio Soares, Marcos Rogério André

**Affiliations:** 1 Vector-Borne Bioagents Laboratory (VBBL), Department of Pathology, Reproduction and One Health, School of Agricultural and Veterinarian Sciences (FCAV), São Paulo State University (UNESP), Jaboticabal 14884-900, SP, Brazil; ana.calchi@unesp.br (A.C.C.);; 2 Laboratório de Protozoologia e Rickettsioses Vetoriais (ProtoZooVet), Faculdade de Veterinária, Universidade Federal do Rio Grande do Sul (UFRGS), Porto Alegre 91540-000, RS, Brazil; joaresmay@gmail.com (J.A.M.-J.); vbagios@gmail.com (V.B.-S.); laurabergervet@gmail.com (L.B.); rafambohn@hotmail.com (R.M.-B.); jfsvet@gmail.com (J.F.S.); 3 National PhD Program in One Health Approaches to Infectious Diseases and Life Science Research, Department of Public Health, Experimental and Forensic Medicine, University of Pavia, 27100 Pavia, Italy; renata.fagundes.mm@gmail.com; 4 Department of Veterinary Medicine, University of Bari “Aldo Moro", 70121 Valenzano, Italy; 5 Laboratório de Mamíferos, Federal University of Paraíba (UFPB), João Pessoa 58051-900, PB, Brazil; laizabraga@gmail.com (L.d.Q.V.B.); lopesrocha.fabiana@gmail.com (F.L.R.); 6 Departamento de Clínica e Cirurgia Veterinária, School of Agricultural and Veterinarian Sciences (FCAV), São Paulo State University (UNESP), Jaboticabal 14884-900, SP, Brazil; murillo_kirnew@yahoo.com.br (M.D.K.); matheusmedvet@gmail.com (M.F.S.); roberto.ampuero@hotmail.com (R.A.N.A.); 7 Intracellular Pathogens Research Laboratory, College of Veterinary Medicine, Comparative Medicine Institute, North Carolina State University, Raleigh, NC 27607, USA; comanvel@ncsu.edu (C.O.M.); ebbreits@ncsu.edu (E.B.B.); rgmaggi@ncsu.edu (R.G.M.); 8 Laboratório de Coleções Zoológicas, Instituto Butantan, São Paulo 05503-900, SP, Brazil; ricardo.bassini@gmail.com; 9 Parasitic Biology Laboratory, Interface Research Group Between Human, Animal and Environmental Health, Universidade Católica Dom Bosco, Campo Grande 79117-900, MS, Brazil; herrera@ucdb.br; 10 Laboratory of Genomics and Molecular Biology, School of Health and Life Sciences, Pontifícia Universidade Católica do Rio Grande do Sul, Porto Alegre 90619-900, RS, Brazil; eduardo.eizirik@pucrs.br

**Keywords:** *Cytauxzoon brasiliensis*, *Cytauxzoon felis*, genovariants, jaguars, ocelots

## Abstract

Domestic and wild felids are frequently parasitized by apicomplexan protozoa in the genus *Cytauxzoon*. Expanding species diversity has recently been described within this genus, with potential implications for epidemiology and pathogenesis. In light of these findings, this study assessed the genetic diversity of *Cytauxzoon* spp. in wild felids (n = 66) from different eco-regions of Brazil and Argentina. Of the 66 blood samples analyzed, 53 (80.3%) were 18S rRNA gene PCR-positive for *Cytauxzoon* spp., including 43 jaguars (*Panthera onca*) and 10 ocelots (*Leopardus pardalis*). *Panthera onca* specimens (100%, 43/43) were most frequently infected, followed by *Leopardus pardalis* (76.9%; 10/13). *Cytauxzoon* spp. were not detected in *Leopardus braccatus* (n = 1) or *Puma concolor* (n = 9). Phylogenetic analyses of fragments of the 18S rRNA, *cytB*, and *cox-1* gene sequences from jaguars were closely related to *Cytauxzoon felis*. In contrast, sequences from ocelots were more closely associated with *Cytauxzoon brasiliensis*. Distance and haplotype analysis further confirmed the circulation of at least two distinct genovariants of *C. felis* among jaguars, as evidenced by their close positioning and low genetic divergence (0–0.14% for 18S rRNA, 0.37–0.56% for *cytB*, and 0.08–0.74% for *cox-1*). Additionally, sequence data from ocelots suggested that multiple genovariants of *C. brasiliensis* are circulating among these cats in different Brazilian eco-regions. Our study provides evidence of two distinct *Cytauxzoon* organisms parasitizing free-ranging and captive jaguars and ocelots, respectively, in Brazil and Argentina.

## 1. Introduction

*Cytauxzoon* spp. (Piroplasmida: Theileriidae) are apicomplexan protozoa that parasitize erythrocytes, monocytes, and macrophages in mammals [1]. This genus was first described in 1948 by Neitz and Thomas, who identified the piroplasmid in the common duiker (*Sylvicapra grimmia*) in South Africa. Since schizonts of the parasite were not found in lymphocytes, as commonly observed in *Theileria* spp., a new genus was established [2,3]. Currently, the genus *Cytauxzoon* has been primarily described in domestic and wild felids [1,3,4,5,6,7], with limited reports in meerkats [8] and bears [9,10].

Six *Cytauxzoon* species have been described in felids to date: *Cytauxzoon felis*, *Cytauxzoon manul*, *Cytauxzoon europaeus*, *Cytauxzoon otrantorum*, *Cytauxzoon banethi*, and *Cytauxzoon brasiliensis* [4,6,7,11]. Phylogenetic inferences based on distinct molecular markers (18S rRNA, *cox-1*, and *cytB*) have identified two major geographically limited clades. The first clade includes *Cytauxzoon* species found in the New World (*C. felis* and *C. brasiliensis*), and the second clade consists of species detected in the Old World (*C. manul* in Asia, and *C. europaeus*, *C. otrantorum*, and *C. banethi* in Europe) [6,7].

Out of six *Cytauxzoon* species described so far, *C. felis* is the most pathogenic species infecting cats. Disease in domestic cats presents with fever, weight loss, lethargy, dehydration, dyspnea, anemia, pale mucous, hemolytic crises, jaundice, and even death [1,12]. In the past, mortality rates in cats in the USA were approximately 100%; however, mortality has decreased with advances in treatment and improved molecular diagnostics for earlier infection detection [12,13]. Disease has been reported in association with *Cytauxzoon*-infected cats in Europe; however, there is no substantial evidence that disease manifestation is related exclusively to *Cytauxzoon* infection [14,15,16,17,18,19]. The outcome of *Cytauxzoon* infection in free-ranging felids is largely unknown, as these animals are exposed to numerous infectious agents. Fatal cytauxzoonosis has been documented in captive lions (*Panthera leo*) [20] and in a jaguar (*Panthera onca*) [21] in Brazil, a captive Bengal tiger (*Panthera tigris*) in Germany [22], and a captive-reared white tiger (*Panthera tigris*) [23] and bobcat (*Lynx rufus*) in the USA [5].

The only experimentally confirmed vectors for *C. felis* are *Amblyomma americanum* and *Dermacentor variabilis*, which are exclusively found in the USA [24,25]. *Amblyomma sculptum* and *Ixodes ricinus* have been suggested as potential vectors for the species of *Cytauxzoon* found in Brazil and Europe, respectively [26,27,28,29].

In South America, descriptions of *Cytauxzoon* spp. have only been published in Brazil, where this piroplasmid has been detected in captive and free-ranging wild felids, including lions, jaguars, pumas (*Puma concolor*), ocelots (*Leopardus pardalis*), and little spotted cats (*Leopardus tigrinus*) [7,20,21,27,30,31,32,33,34,35], as well as in domestic cats [36,37,38,39,40,41,42]. The majority of published studies were based on a short fragment of the 18S rRNA, precluding accurate phylogenetic assessment of the isolates that circulate among wild and domestic felids in the country. Using phylogenetic inferences based on the near-complete 18S rRNA and *cythocrome B* (*cytB*) genes, Duarte et al. (2024) [7] demonstrated the occurrence of a novel species (*C. brasiliensis)* in a little spotted cat sampled in central-western Brazil, suggesting a greater diversity of *Cytauxzoon* spp. in South America than previously documented. The purpose of this study was to analyze the genetic diversity of *Cytauxzoon* spp. in jaguars, pumas, and ocelots sampled in different eco-regions of Brazil and Argentina.

## 2. Materials and Methods

### 2.1. Sampling

**Figure 1:**
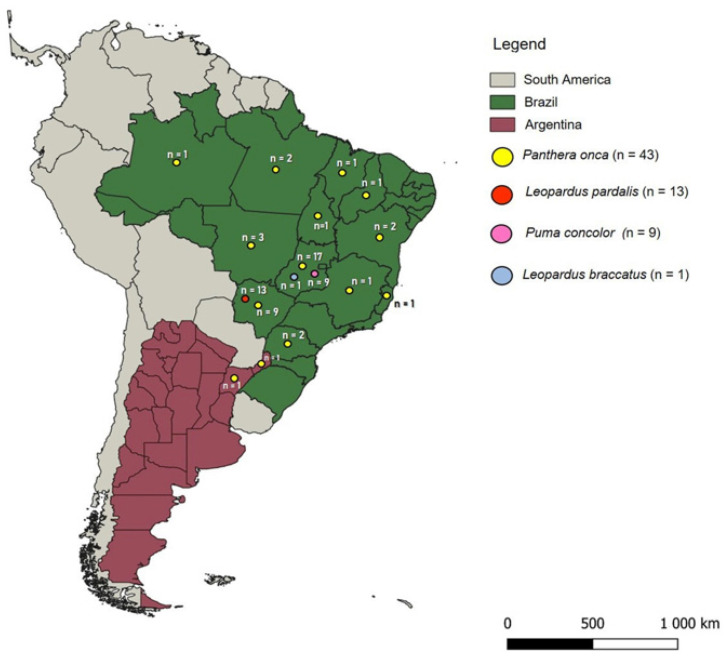
Map of South America illustrating the ecoregions where the free-living or captive felids were sampled.

Between 2015 and 2022, blood samples were collected from 66 wild felids (40 free-ranging and 26 captive felids), including *L. pardalis* (n = 13), *Leopardus braccatus* (n = 1), *P. concolor* (n = 9), and *P. onca* (n = 43). Cats were captured or maintained in different Brazilian states (the states of Amazonas, Bahia, Espírito Santo, Goiás, Maranhão, Mato Grosso, Mato Grosso do Sul, Minas Gerais, Paraná, Piauí, and Tocantins) and Argentina (the provinces of Misiones and Corrientes) (Figure 1). All sampled animals except the ocelots were part of conservation projects. Captures were performed in different ecoregions of Brazil, such as the Cerrado, Pantanal, Amazon Rainforest, and Atlantic Rainforest. Blood samples were collected by puncturing the cephalic or jugular veins using tubes containing EDTA (ethylenediamine tetra acetic acid) and stored at –20 °C until analysis. Samples, collected by different research groups, were provided for molecular screening. The table provided in Supplementary Table S1 shows the collection sites, sample origin, and those institutions responsible for collecting each sample used in this study.

Free-ranging ocelots (*L. pardalis*) were captured in box traps (90 × 45 × 50 cm; Equipos Fauna^®^, Criciúma, SC, Brazil) and sedated with zoletil 50 (8 mg/kg) or a combination of ketamine (15 mg/kg), xylazine (0.7 mg/kg), and midazolam (0.5 mg/kg). The free-ranging large wild felids were captured as described by May-Junior et al. (2021) [43] and Fagundes-Moreira et al. (2022) [27]. After their complete recovery from sedation, all animals were released at the capture sites. For the captive wild felids from the “Instituto Onça Pintada” (IOP), collected by the FCAV/UNESP research group, chemical restraint was performed using an intramuscular dart with a combination of medetomidine (0.08–0.1 mg/kg) and ketamine (5 mg/kg). After sample collection, anesthesia was reversed with atipamezole (0.25 mg/kg), and the animals were placed back in their enclosures.

### 2.2. DNA Extraction and PCR Assays

DNA was extracted from 200 μL of each blood sample using the DNeasy Blood & Tissue Kit (Qiagen, Valencia, CA, USA), following the manufacturer’s instructions. The DNA samples were then subjected to a conventional PCR (cPCR) assay, targeting the mammalian endogenous glyceraldehyde-3-phosphate dehydrogenase (*gapdh*) gene, to confirm the presence of amplifiable DNA [44]. Only the *gapdh*-positive samples were subsequently tested for *Cytauxzoon* spp. DNA. DNA samples were registered under SISGEN #A9CCAD1 (“Sistema Nacional de Gestão do Patrimônio Genético e do Conhecimento Tradicional Associado”).

For *Cytauxzoon* spp. DNA detection, a PCR assay was performed based on the amplification of a 284 bp fragment of the 18S rRNA gene [45]. PCR-positive samples were further characterized using conventional or nested PCR assays targeting different molecular markers: the near-complete 18S rRNA gene (~1500 bp) [46,47,48], *cytB* (~1444 bp) [49], *cox-1* (~1656 bp) [6,50,51], *cox-3* (~600 bp) [51,52], and the 18S–5.8S rRNA (ITS-1) and 5.8S–28S rRNA (ITS-2) regions [53].

The assays were performed using 5 μL of DNA in a reaction mixture containing 0.75 U Platinum Taq DNA Polymerase (Invitrogen, Carlsbad, CA, USA), 10X PCR buffer (100 mM Tris-HCl, pH 9.0, 500 mM KCl), 0.2 mM of each deoxynucleotide (dATP, dTTP, dCTP, and dGTP) (Invitrogen, Carlsbad, CA, USA), 1.5 mM magnesium chloride (Invitrogen, Carlsbad, CA, USA), 0.5 μM of each primer (Invitrogen, Carlsbad, CA, USA), and sterile ultrapure water (Invitrogen, Carlsbad, CA, USA) to a total volume of 25 μL. For the nested PCR assays, 1 μL of the amplified product from the first PCR reaction was used as the target DNA in the second reaction. A DNA sample obtained from a cat that was naturally infected with a Brazilian *Cytauxzoon* sp. [39] and sterile ultrapure water (Life Technologies^®^, Carlsbad, CA, USA) were used as the positive and negative controls, respectively. All primer sequences and thermal conditions used in the PCR assays are described in Supplementary Table S2.

PCR products were separated by horizontal electrophoresis on 1% agarose gel stained with ethidium bromide (Life Technologies™, Carlsbad, CA, USA) in TEB running buffer (pH 8.0) at 100 V/150 mA for 50 minutes. The gels were examined under ultraviolet light using the ChemiDoc MP Imaging System (Bio-Rad, Hercules, CA, USA) and photographed with Image Lab Software v.4.1 (Bio-Rad, Hercules, CA, USA). The amplified products were purified using the Wizard SV Gel and PCR Clean-Up System (Promega, Madison, WI, USA) and bidirectionally sequenced by Sanger’s method using the BigDye Terminator v3.1 Cycle Sequencing Kit (Thermo Fisher Scientific, Waltham, MA, USA) and the ABI 3730 DNA Analyzer (Applied Biosystems, Foster City, CA, USA).

To obtain higher-quality sequences, internal primers were designed for the 18S rRNA, *cytB*, and *cox-1* genes, as follows: 18S-R (5’-TTGAGTCAAATTAAGCCGCA-3’) [54], CytB-F1 (5′-TTGTTTTATTGGGCTGCTGA-3′—used in the jaguar samples), CytB-F2 (5′-GAGTTATTGGGGAGCAACAG-3′—used in the ocelot samples), Cytaux_cytB_Finn (5’-ACCTACTAAACCTTATTCAAGCRTT-3’) [6], and COI-F (5′-TGCTGGTATTGCTAGTGCTT-3′). These primers were only used for sequencing.

### 2.3. Cloning and Sequencing of the Near-Complete 18S rRNA Gene

Three near-complete 18S rRNA amplicons obtained from ocelot blood samples were cloned, due to the presence of multiple peaks in the electropherograms. These amplicons were subjected to pGEM-T Easy (Promega^®^, Madison, WI, USA) cloning, following the manufacturer’s instructions. At least four clones were selected from each positive sample using the blue/white colony-screening method. Colonies containing the gene fragment of interest, as confirmed by PCR, were subjected to plasmid DNA extraction using the Wizard^®^ Plus SV Miniprep DNA Purification System (Promega, Madison, WI, USA). The purified plasmids were then sequenced using the M13 F (5′-CGCCAGGGTTTTCCCAGTCACGAC-3′) and M13 R (5′-GTCATAGCTGTTTCCTGTGTGA-3′) primers [55], which flank the multiple cloning sites of the pGEM-T Easy plasmid, allowing sequencing of the inserted gene fragments. Additionally, a set of internal primers (forward: 5’- CGCGTAAATTACCCAATCCT-3’ and reverse: 5’-TATGGTTAGGACTACGACGG-3’) designed for the sequences of this study were also used to obtain higher-quality sequences. Sequencing was carried out as previously described.

### 2.4. BLASTn, Phylogenetic, and Haplotype Analyses

The quality of the bidirectional sequences and the consensus sequences were analyzed and obtained using Phred-Phrap software (version 23) [56,57]. The BLASTn program (available online: https://blast.ncbi.nlm.nih.gov/Blast.cgi accessed on 26 November 2024) [58] was used to compare the obtained sequences with those previously deposited sequences in the GenBank database [59]. The sequences, saved in “FASTA” format, were aligned with homologous sequences retrieved from GenBank using the MAFFT software [60] and trimmed with Bioedit v. 7.0.5.3 [61]. For the *cox-1* gene, the amino acid sequences were used for alignment and subsequent phylogenetic analysis. The W-IQ-Tree software was employed to choose the evolutionary model based on the BIC criterion and for phylogenetic analysis using the maximum likelihood method (available online: http://iqtree.cibiv.univie.ac.at/ accessed on 26 November 2024) [62]. The phylogenetic trees were edited using Treegraph 2.0.56-381 beta software [63].

The same alignments, excluding the outgroups, were used to construct genealogies via network analysis with the neighbor-net method, applying 100 bootstrap replicates using the Splitstree v4.11.3 software [64]. Additionally, a pairwise distance matrix was calculated using the *p*-distance method with MEGA X software [65,66,67].

New alignments were constructed using the near-complete 18S rRNA, *cytB*, and *cox-1* nucleotide sequences of *Cytauxzoon* obtained in the present study, along with the phylogenetically closest sequences in GenBank. These alignments were used in the genetic diversity analysis, conducted with DnaSP v5 software, to calculate nucleotide diversity (π), haplotype diversity (Hd), the number of haplotypes (h), and the average number of nucleotide differences (K) [68]. The haplotype network was constructed using population analysis with reticulate trees (popART) software [69], applying the TCS network method [70].

## 3. Results

All 66 blood DNA extractions were endogenous mammalian *gapdh* gene PCR-positive, of which 53 (80.3%) tested positive for *Cytauxzoon* spp., based on amplification of the 18S rRNA gene. All *P. onca* (43/43) specimens and 76.9% of *L. pardalis* specimens were 18S rRNA PCR-positive for *Cytauxzoon.* No *L. braccatus* or *P. concolor* felids were found to be positive for *Cytauxzoon* spp. Of the 53 short-fragment PCR-positive samples, readable sequences were obtained from 51 (96.2%) of the *Cytauxzoon* 18S rRNA gene (1500 bp), 50 (94.3%) of the *cox-1* and *cytB Cytauxzoon* spp. genes, and 48 (90.6%) of the ITS-1 and ITS-2 regions of *Cytauxzoon* spp. There was no amplification of the *cox-3* gene. Table 1 summarizes the number of *Cytauxzoon* PCR-positive samples for each wild felid species and sampling location.

A subset of conventional PCR-positive samples was selected for DNA sequencing based on band quality (single, clear, and intense bands on agarose gel electrophoresis). High-quality consensus sequences were deposited in GenBank: the 18S rRNA gene (18 from jaguars and 3 from ocelots, with the latter cloned to yield 11 sequences; accession numbers PQ686785–PQ686813), *cytB* gene (20 from jaguars and 4 from ocelots; GenBank accession numbers PQ724014–PQ724037), *cox-1* gene (13 from jaguars and 1 from an ocelot; GenBank accession numbers PQ724000–PQ724013), and the ITS-1 (GenBank accession numbers PQ687019 and PQ687020) and ITS-2 regions from ocelots (GenBank accession numbers PQ687021 and PQ687022).

Based upon BLASTn analysis, the 18S rRNA sequences (~1500 bp) obtained from jaguar DNA blood samples were 99.78–99.86% identical to *C. felis* sequences from domestic cats from the USA (AF399930), while those from ocelots were 99.87–99.94% identical to *Cytauxzoon* sp. obtained from the ocelots from Brazil (GU903911). The *cytB* sequences from the jaguars exhibited 99.52%–99.56% identity with *C. felis* samples from a cat from the USA (KC207821), while those from ocelots were 99.03%–99.12% identical with *C. brasiliensis* detected in *L. tigrinus* samples from Brazil (PP588457). The *cox-1* sequences from the jaguars showed 99.42%–99.92% similarity with *C. felis* sequences from cats from the USA (MT916252), while the sequences obtained from ocelots showed 91.46% similarity with *C. felis* from domestic cats (MT916250). The ITS-1 sequences from ocelots were identical to a *Cytauxzoon* sp. sequence obtained from a domestic cat from Brazil (KP683154), while the ITS-2 sequences were 94.98 - 95.82% similar to a *Cytauxzoon* sp. sequence amplified from an ocelot from Brazil (FJ876458) (Supplementary Materials Table S3).

**Figure 2:**
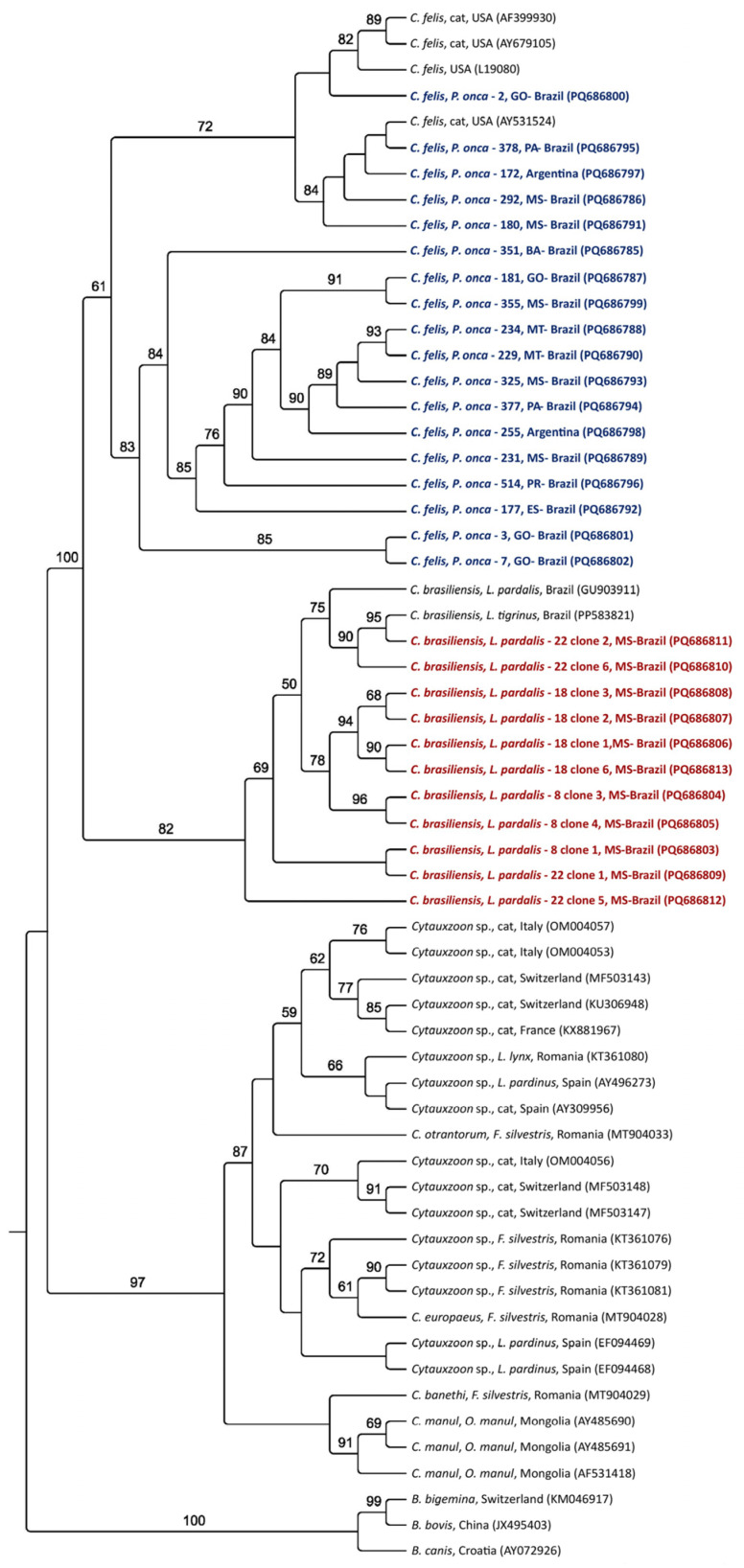
Phylogenetic analysis of *Cytauxzoon* spp. The 18S rRNA sequences from an alignment of 1587 characters were generated by maximum likelihood and with the TN+G evolutionary model. *B. bigemina, B. bovis,* and *B. canis* were used as an outgroup. Sequences obtained from jaguar DNA blood samples are in blue, whereas those obtained from ocelot DNA are in red.

**Figure 3:**
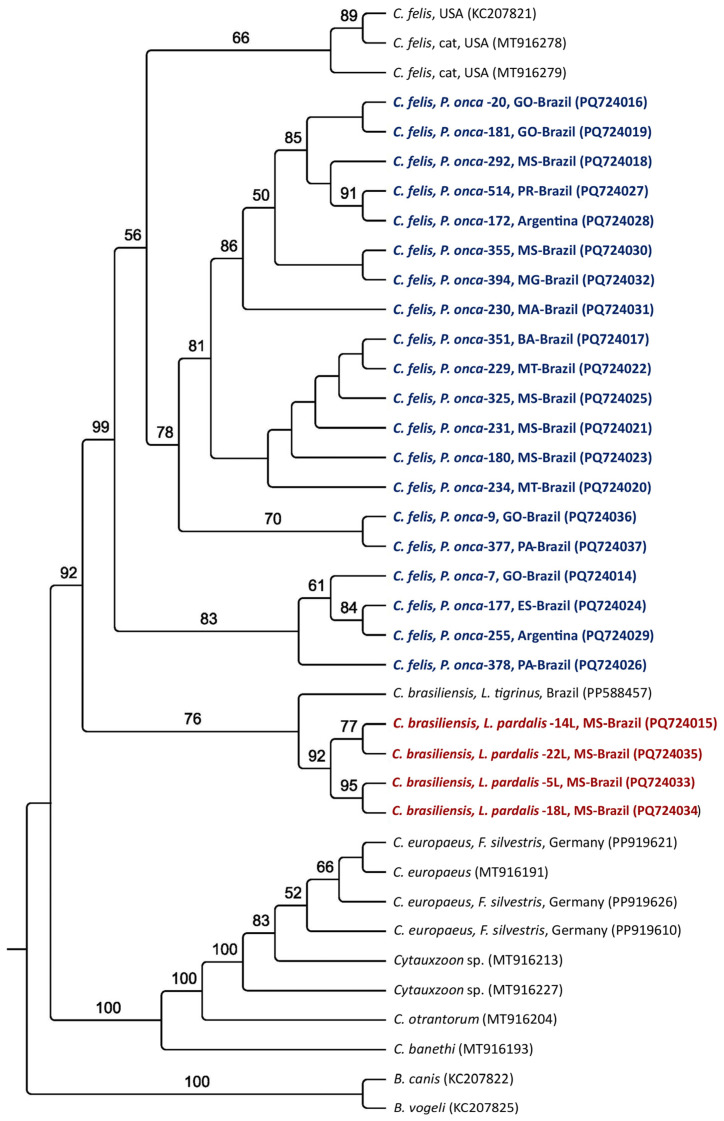
Phylogenetic analysis of *Cytauxzoon* spp. The *cytB* sequences from an alignment of 1082 bp were generated by maximum likelihood and with the K3Pu+G evolutionary model. *B. canis* and *B. vogeli* were used as an outgroup. Sequences obtained from jaguar DNA blood samples are in blue, whereas those obtained from ocelots are in red.

**Figure 4:**
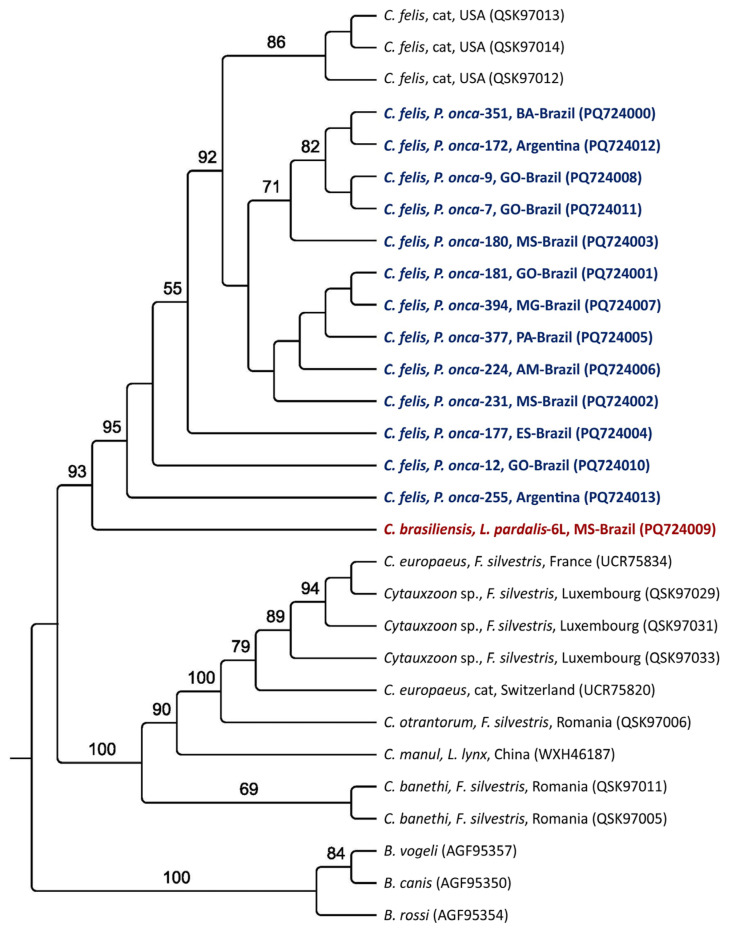
Phylogenetic analysis of *Cytauxzoon* spp. COX-1 amino acid sequences from an alignment of 417 amino acids were generated by maximum likelihood and with the mtZOA+I evolutionary model. *B. canis*, *B. vogeli*, and *Babesia rossi* were used as an outgroup. The sequences obtained from jaguar DNA blood samples are in blue, whereas those obtained from ocelot DNA are in red.

**Figure 5:**
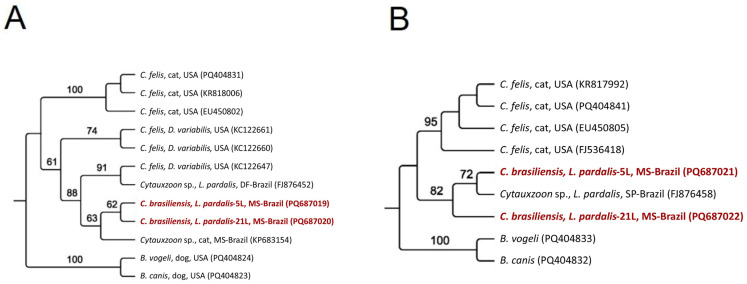
(**A**). Phylogenetic analyses of *Cytauxzoon* spp. ITS-1 sequences from an alignment of 558 bp were generated by maximum likelihood and with the K2P evolutionary model. (**B**). Phylogenetic analyses of *Cytauxzoon* spp. ITS-2 sequences were from an alignment of 252 characters generated by maximum likelihood and with the HKY evolutionary model. *Babesia canis* and *B. vogeli* were used as an outgroup. The sequences obtained from ocelot DNA samples are in red.

Maximum likelihood phylogenetic analyses of all molecular markers grouped the sequences obtained from jaguars into subclades closer to the *C. felis* clade from the USA. All sequences from ocelots formed a single clade, sister to the main clade of *C. felis* and jaguars, along with *C. brasiliensis*; all were supported by high bootstrap values (Figure 2, Figure 3, Figure 4 and Figure 5). Based on the 18S rRNA gene phylogenetic inference (alignment of 1587 characters in the evolutionary model TN+G), six sequences obtained from jaguars (#2, #378, #172, #292, #180, and #351) clustered with *C. felis,* while the other 13 sequences obtained from these felid species formed a sister clade to that of *C. felis* (Figure 2). In contrast, phylogenies based on all *cytB* (alignment of 1082 bp, evolutionary model K3Pu+G) (Figure 3) and COX-1 (alignment of 417 aa; evolutionary model mtZOA+I) (Figure 4) genes clearly clustered with jaguar-associated *C. felis* sequences. The phylogeny based on the ITS-1 intergenic region (alignment of 558 bp; evolutionary model K2P) and ITS-2 (alignment of 252 bp; evolutionary model HKY) grouped the ocelot sequences with a sequence previously amplified from a sample taken from an ocelot in Brazil (Figure 5A,B).

**Figure 6:**
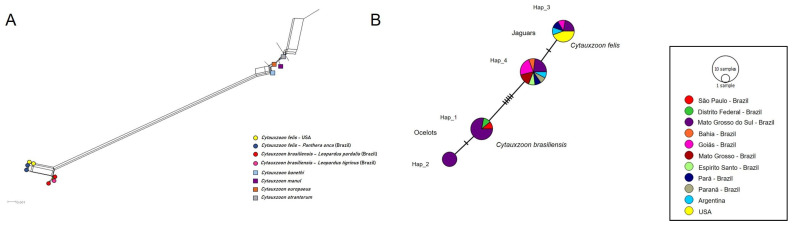
(**A**). *Cytauxzoon* split-network distance analysis of 18S rRNA sequences. (**B**). The TCS network formed among 18S rRNA sequences (1375 bp), as detected in wild and domestic cats. The size of the circles varies according to the number of sequences belonging to each haplotype. The color represents the geographic location where each sequence was detected. The black vertical lines represent the mutational events that occurred between each haplotype and the black circles represent median vectors.

**Figure 7:**
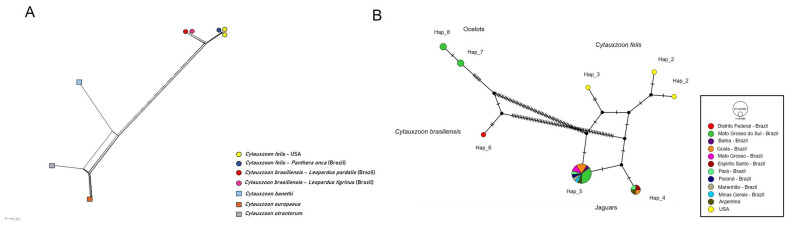
(**A**). *Cytauxzoon* split-network distance analysis of *cytB* sequences. (**B**). The TCS network formed among *cytB* sequences (1027 bp), as detected in wild and domestic cats. The size of the circles varies according to the number of sequences belonging to each haplotype. The color represents the geographic location where each sequence was detected. The black vertical lines represent the mutational events that occurred between each haplotype and the black circles represent median vectors.

**Figure 8:**
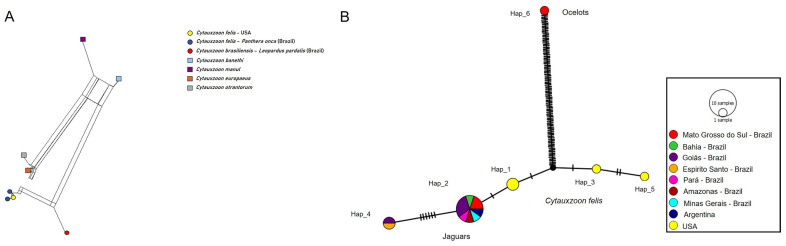
(**A**). *Cytauxzoon* split-network distance analysis of COX-1 sequences. (**B**). The TCS network formed among cox-1 sequences (1219 bp), as detected in wild and domestic cats. The size of the circles varies according to the number of sequences belonging to each haplotype. The color represents the geographic location where each sequence was detected. The black vertical lines represent the mutational events that occurred between each haplotype and the black circles represent median vectors.

Distance analyses of the 18S rRNA (Figure 6A), *cytB* (Figure 7A), and COX-1 (Figure 8A) revealed a clear separation between the jaguar and ocelot sequences. The jaguar sequences were much closer to *C. felis*, while the ocelot sequences were nearer to *C. brasiliensis*. In addition, there is a clear relationship between the sequences detected in jaguars and *C. felis,* and a greater separation between the other *Cytauxzoon* species. In pairwise *p*-distance analyses, the divergence between *C. felis* and the jaguar 18S rRNA sequences ranged from 0 to 0.14%, that between *C. brasiliensis* and ocelots ranged from 0 to 0.07%, and that between the ocelot and jaguar sequences ranged from 0.34% to 0.51% (Supplementary Materials Table S4). For *CytB*, the divergence between *C. felis* and jaguar sequences ranged from 0.37% to 0.56%, from 0.88% to 0.97% between *C. brasiliensis* and ocelot sequences, and from 7.27% to 7.43% between the jaguar and ocelot sequences (Supplementary Materials Table S5). For *cox-1*, the divergence between the *C. felis* and jaguar sequences ranged from 0.08% to 0.74%, while the divergences between the jaguar and ocelot sequences ranged from 8.59% to 8.69% (Supplementary Material Table S6). No *cox-1* sequences for *C. brasiliensis* were available in GenBank.

Haplotype analysis of 35 18S rRNA sequences identified 4 distinct haplotypes among the ocelots: Haplotype #1 was formed by seven sequences drawn from ocelots in this study, a sequence from an ocelot from São Paulo, Brazil, and *C. brasiliensis*; Haplotype #2 comprised four cloned sequences obtained from an ocelot (ID #18); Haplotype #3 included five sequences from jaguars and *C. felis* sequences from the USA; and Haplotype #4 consisted of 13 sequences from jaguars. All haplotypes were separated from each other by mutational events, without median vectors (inferred ancestral nodes). Five mutational events separated *C. felis* and the jaguar-associated haplotypes from *C. brasiliensis* and ocelot-associated haplotypes (Figure 6B).

The analysis of 29 *cytB* sequences identified 8 haplotypes: Haplotypes #1 to #3 were formed by *C. felis* sequences; Haplotypes #4 and #5 by four and 17 jaguar sequences, respectively; Haplotype #6 by a *C. brasiliensis* sequence; and Haplotypes #7 and #8 by two sequences each from ocelots in this study. While *C. felis* and the jaguar-associated haplotypes were separated by median vectors and few mutational events, *C. felis* and jaguar-associated haplotypes were separated from *C. brasiliensis* and ocelot-associated haplotypes were separated by median vectors and several mutational events. *Cytauxzoon brasiliensis* and the ocelot-associated haplotypes were separated by median vectors and few mutational events (Figure 7B). The haplotype network of the *cox-1* gene, based on 17 sequences, formed 6 haplotypes: Haplotypes #1, #3, and #5 comprised only *C. felis* sequences; Haplotypes #2 and #4 comprised 10 and 2 jaguar-associated sequences, respectively; and Haplotype #6 was an ocelot-associated sequence. A single median vector grouped the haplotypes into clusters (#1, 2, and 4; #3 and 5; #6), separated by mutational events. While few mutational events separated *C. felis* and the jaguar-associated haplotypes, the ocelot-associated haplotype was separated from *C. felis* and the jaguar-associated haplotypes by a median vector and several mutational events (Figure 8B). Table 2 shows the genetic diversity values for each analyzed gene.

## 4. Discussion

In this study, all 43 jaguars tested were infected with a *Cytauxzoon* spp., corroborating previous reports carried out in Brazil. Previously, high molecular positivity for this protozoan in jaguars (96.5–100%), along with the persistence of positivity after recapture, suggested that this wild felid species might play a role as a reservoir host [27,32]. Additionally, consistent with the present study, which found a high occurrence (76.9% [10/13]) in ocelots, Fagundes-Moreira et al. (2022) [27] reported 80% (4/5) positivity in ocelots sampled in midwestern Brazil. Interestingly, no ocelots sampled in the state of Rio Grande do Sul State tested *Cytauxzoon* PCR-positive [27]. Contrary to previous reports, we did not find any pumas infected with a *Cytauxzoon* spp. [27,30,35], perhaps due to the limited sampling numbers (n = 9).

It has been previously established that the haplotypes of *Cytauxzoon* spp. circulating in domestic and wild felids from Brazil are phylogenetically related to *C. felis* from the USA [32,33,34,37,39,40,42,71,72]. However, as most of these studies used only a short fragment of the 18S rRNA gene, the exact phylogenetic position remained uncertain. Additionally, domestic cats in Brazil do not exhibit the severe clinical signs commonly observed in cats from the USA [37,39,40] and, to date, schizonts have never been demonstrated in *Felis catus* in the Brazilian territory. In a recent study, a new species (*C. brasiliensis*) was described in a little spotted cat specimen from Brazil. This new species, molecularly identified using the near-complete 18S rRNA and *cytB* genes, forms a distinct clade, sister to the *C. felis* clade from the USA [7]. Our study documented the presence of *C. brasiliensis* in ocelots from Mato Grosso do Sul state, Brazil, and also detected haplotypes related to *C. felis* in jaguars.

Interestingly, one of the jaguars sampled in Argentina that tested positive for *C. felis* was born in captivity after the crossbreeding of two animals from Brazil. Although one study failed to demonstrate the occurrence of perinatal infection by *Cytauxzoon* sp. in domestic cats [73], other studies have reported the presence of *Cytauxzoon* spp. in domestic kittens and wild felid kittens [5,17,20,74]. However, none of the studies have confirmed whether this was due to vertical transmission or tick infestation. In this study, we were unable to determine whether the infection resulted from perinatal transmission or from the presence of tick vectors in the animal’s enclosure in Argentina. 

In this study, phylogenetic analysis based on the near-complete 18S rRNA and *cytB* genes documented a clear separation between the sequences obtained from ocelots, which were grouped within the *C. brasiliensis* clade, and those from jaguars, which clustered into subclades within the *C. felis* clade. These findings demonstrate the presence of at least two distinct *Cytauxzoon* species parasitizing free-ranging and captive felids in Brazil. Phylogenetic analysis based on the translated COX-1 protein sequence also separated ocelot and jaguar-associated *C. felis* sequencesinto separate clades. However, the lack of *C. brasiliensis cox-1* sequences in GenBank and the limited number of readable sequences obtained in this study hampered further interpretation of phylogenetic inferences based on this molecular marker.

In addition, phylogenetic analysis of 13 near-complete 18S rRNA gene sequences from jaguars identified a sister clade to the *C. felis* clade detected in the USA, whereas 5 sequences were clustered within the *C. felis* clade. However, in the *cytB* and COX-1-based phylogenetic analyses, all jaguar-associated sequences formed a single clade, distinct from the *C. felis* clade from the USA. Panait et al. (2021) [6] described three new *Cytauxzoon* species (*C. europaeus*, *C. otrantorum*, and *C. banethi*) circulating in Europe, but the 18S rRNA gene alone was insufficient to differentiate these species, which did not reveal high genetic divergence. In contrast, due to their greater genetic variability, two mitochondrial genes (*cytB* and *cox-1*) more effectively differentiated these three species, highlighting the limitations of the 18S rRNA gene for distinguishing closely related taxa [6,51].

To assess whether the *Cytauxzoon* spp. infecting jaguars represent distinct haplotypes of *C. felis* or a possibly new species, distance and haplotype analyses based on three distinct molecular markers (near-complete 18S rRNA, *cytB*, and *cox-1*) were analyzed. The genealogies constructed using the neighbor-net method indicate that *C. felis* is closely related to the jaguar-associated *C. felis*, while the separation among other *Cytauxzoon* species is more pronounced. Pairwise *p*-distance analyses revealed that the divergence between the jaguar-associated *Cytauxzoon* sp. and *C. felis* was 0 to 0.14% for the near-complete 18S rRNA, 0.37 to 0.56% for *cytB*, and 0.08 to 0.74% for *cox-1*. In comparison, the divergence between *C. brasiliensis* and *C. felis* was approximately 0.4% for the 18S rRNA and 7% for *cytB* genes, while interspecific divergence among *C. europaeus*, *C. otrantorum*, and *C. banethi* was approximately 0.1% (18S rRNA) [6,7], 9 to 22% (*cytB*), and 6 to 14% (*cox-1*), values that are much higher than those found in this study. These results indicate infection with *C. felis* genovariants in jaguars rather than a novel *Cytauxzoon* species.

Haplotype analyses based on the *cytB* and *cox-1* genes indicated that the *Cytauxzoon* spp. sequences from jaguars were divided into two distinct haplotypes, although none of them were shared with *C. felis* from the USA. In contrast, the 18S rRNA-based haplotype analysis indicated that some jaguar-associated haplotypes belonged to the same haplotype as *C. felis*, likely due to the high conservation of this molecular marker, which can make accurate analysis of genetic diversity difficult, as previously reported by Panait et al. (2021) [6]. In contrast, the haplotype analyses based on *cytB* and *cox-1* genes were consistent with one another, each identifying two haplotypes.

Regarding the ocelot-associated *Cytauxzoon* spp. sequences, two haplotypes were found for both the 18S rRNA and *cytB* genes. Only one sequence was obtained for *cox-1,* negating the phylogenic analysis. While one of the 18S rRNA haplotypes was shared with *C. brasiliensis*, the *cytB* haplotypes detected in ocelots were separated from *C. brasiliensis* by median vectors, indicating the occurrence of distinct genovariants of this recently described *Cytauxzoon* species. Further studies are needed to fully explore the genetic diversity of *C. brasiliensis* in Brazil.

The ITS region has been used to demonstrate high *C. felis* genotypic diversity in cats from the USA [75,76,77,78,79]. Two complete ITS-1 and ITS-2 intergenic region sequences were obtained from the *Cytauxzoon* spp. infecting two ocelots in this study. Phylogenetic analysis based on the ITS-2 region positioned both sequences in a clade with another haplotype that was previously detected in an ocelot from Brazil. The ITS-1-based phylogenetic analysis grouped both the sequences detected in ocelots in this study and sampled in the state of Mat Grosso do Sul (MS) with a sequence previously amplified from a domestic cat, also from MS. These findings indicate that *C. brasiliensis* can infect little spotted cats, ocelots, and domestic cats in Brazil. 

Given the known occurrence of distinct *Cytauxzoon* species in Brazil, including genovariants of *C. felis*, further studies are needed to determine which species commonly infect domestic cats and to identify the primary tick vectors. Although molecular and epidemiological evidence suggests *A. sculptum* as a putative tick vector for *Cytauxzoon* spp. in Brazil [27,28], experimental studies are necessary to assess vector competence. To date, fatal cases of *Cytauxzoon* spp. infection with the presence of schizonts in several tissues have been reported in wild felids [20,21], but not in domestic cats. Additional research is required to understand whether the circulating *Cytauxzoon* spp. haplotypes in Brazil are non-pathogenic or whether pathogenicity is only associated with stress, debilitation, or poor immunity [20,21]. Finally, considering that at least two *Cytauxzoon* species can be found in Brazil, future studies should utilize the near-complete 18S rRNA and/or mitochondrial genes when investigating the phylogenetic positioning of these parasites in domestic and wild felids. Whether *C. brasiliensis* infects felids in Argentina and other South American countries is yet to be determined.

## 5. Conclusions

This study documented the presence of at least two distinct *Cytauxzoon* species in wild felids from South America: while *C. brasiliensis* was detected in ocelots from Brazil, genovariants of *C. felis* were detected in jaguars from Brazil and Argentina. 

## Figures and Tables

**Table 1:** Positivity of molecular tests for *Cytauxzoon* using different molecular markers in blood samples collected from free-ranging or captive felids from different eco-regions of Brazil and Argentina. Brazilian states where felids were sampled: MS = Mato Grosso do Sul, GO = Goiás State, MT = Mato Grosso State, MG = Minas Gerais State, ES = Espírito Santo State, PR = Paraná State, PA = Para State, PI = Piauí State, BA= Bahia State, MA = Maranhão State, AM = Amazonas State, and TO = Tocantins State.

Wild Felids Species	Collection site (Number of Samples)	Positive for *Cytauxzoon* sp.	Near-complete 18S rRNA (~1500 bp)	*cytB* (~1444 bp)	*cox-1* (~1656 bp)	ITS-1	ITS-2
*Leopardus pardalis*	MS (n = 13)	10	9	10	10	10	10
*Leopardus braccatus*	GO (n = 1)	0	-	-	-	-	-
*Panthera onca* (n = 43)	MS (n = 9)	9	9	9	9	9	9
	MT (n = 3)	3	3	3	3	3	3
	GO (n = 17)	17	16	15	14	13	13
	MG (n = 1)	1	1	1	1	1	1
	ES (n = 1)	1	1	1	1	1	1
	PR (n = 2)	2	2	2	2	1	1
	PA (n = 2)	2	2	2	2	2	2
	PI (n = 1)	1	1	1	1	1	1
	BA (n = 2)	2	2	2	2	2	2
	MA (n = 1)	1	1	1	1	1	1
	AM (n = 1)	1	1	0	1	1	1
	TO (n = 1)	1	1	1	1	1	1
	Argentina (n = 2)	2	2	2	2	2	2
*Puma concolor*	GO (n = 9)	0	-	-	-	-	-
Total	66	53	51	50	50	48	48

**Table 2:** Genetic diversity and polymorphisms of *Cytauxzoon* 18S rRNA, *cytB*, and *cox-1* sequences.

Gene	Bp	N	VS	GC%	h	Hd (mean ± SD)	π (mean ± SD)	K
18S rRNA	1375	35	7	43.4	4	0.738 ± 0.033	0.00219 ± 0.00019	3.00504
*cytB*	1027	29	83	29.7	8	0.645 ± 0.095	0.02242 ± 0.00659	23.02709
*cox-1*	1219	17	110	30.9	6	0.654 ± 0.122	0.01180 ± 0.00853	14.38235

N = number of sequences analyzed; VS = number of variable sites; GC% = G+C content; h = number of haplotypes; Hd = haplotype diversity; SD = standard deviation; π = nucleotide diversity (per site); K = nucleotide difference number.

## Data Availability

The datasets generated and analyzed during the current study are available on the NCBI Genbank Nucleotide platform (https://www.ncbi.nlm.nih.gov/genbank/, accessed on 30 December 2024) and can be accessed through accession numbers: PQ686785–PQ686813 for the 18S rRNA gene; PQ724000–PQ724013 for the *cox-1* gene; PQ724014–PQ724037 for the *cytB* gene; PQ687019 and PQ687020 for the internal transcribed spacer 1 (ITS-1); PQ687021 and PQ687022 for the internal transcribed spacer 2 (ITS-2).

## Supplementary Materials

The following supporting information can be downloaded at: https://www.mdpi.com/article/10.3390/pathogens14020148/s1, Tabe S1: Description of the locality, origin, and institutions responsible for sample collec-tion; Table S2: Description of the primers, amplicon sizes, and thermal conditions used in conventional and nested PCR assays for the detection and characterization of *Cytauxzoon* spp.; Table S3: BLASTn results of the sequences obtained in the amplification of five distinct molecular markers from *Cytauxzoon* spp. from wild felid blood samples; Table S4: Pairwise analysis of the near-complete 18S rRNA sequences; Table S5: Pairwise analysis of the near-complete *cytB* sequences; Table S6: Pairwise analysis of the near-complete *cox-1* sequences.
