# Supplementary material for: Diversity of Cytauxzoon spp. (Piroplasmida: Theileriidae) in Wild Felids from Brazil and Argentina
Source: Pathogens. 2025 Feb 4;14(2):148. doi: 10.3390/pathogens14020148 (PMC12184619; doi:10.3390/pathogens14020148)
Supplement: Supplementary file 1 [file pathogens-14-00148-s001.zip › Supplementary material_2.pdf]

Table S2. Description of primers, amplicon sizes and thermal conditions used in conventional and nested PCR assays for detection and characterization of *Cytauxzoon* spp.

Agent	Gene/ name primer	Primers sequences	Size (bp)	Thermal sequences	Reference
**Endogenous gene**	*gapdh*<br>-GAPDHF<br>-GAPDHR	5′-TCCAACACCACCACTGAGATCGGAC -3′<br>5′- GTGAGAAGAAATCGGACTGGCC-3′	400	95°C for 5 min;<br>35 cycles: 95°C for 15s, 50°C for 30s and 72°C for 30s;<br>72ºC for 5 min	[44]
***Cytauxzoon* spp. (screening)**	18S rRNA<br>-CytF<br>-CytR	5′-GCGAATCGCATTGCTTTATGCT-3′<br>5′-CCAAATGATACTCCGGAAAGAG-3′	300	95°C for 5 min;<br>40 cycles: 95°C for 45s, 59°C for 45s and 72°C for 1 min;<br>72°C for 5 min	[45]
**Piroplasmids (characterization)**	18S rRNA<br>-Nbab_1F<br>-18SApiR	5′-AAGCCATGCATGTCTAAGTATAAGCTTTT-3′<br>5′-GGATCACTCGATCGGTAGGAG-3′	1500	95°C for 5 min;<br>50 cycles: 95°C for 30s, 60°C for 30s and 72°C for 2min;<br>72°C for 5 min	[48]
**Piroplasmids (characterization)**	18S rRNA<br>-NBAB1Bab<br>-Hep1615R	5′-GGATAACCGTGC TAATTGT-3′<br>5′-AAAGGGCAGGGA CGTAATC-3′	1500	95°C for 10 min;<br>40 cycles: 95°C for 30s, 55°C for 30s and 72°C for 90s;<br>72°C for 10 min	[46,47]
***Cytauxzoon* spp. (characterization)**	*cytB*<br>-Cytaux_cytB_F1<br>-Cytaux_cytb_R3	5′-CTTAACCCAACTCACGTACC-3′<br>5′- GGTTAATCTTTCCTATTCCTTACG-3′	1434	95ºC for 3 min;<br>36 cycles; 95ºC for 30s, 53ºC for30s and 72ºC for 1 min<br>72ºC for 7 min	[49]
***Cytauxzoon* spp. (characterization-nPCR)**	*cox-1*<br>1st round<br>-Th-for2<br>-Piro mt R1<br>2nd round<br>-Th-For2<br>-Cytaux 260R	5′- TGGYTKGCTTATTGGTTTGG-3′<br>5′- ACTTTGAACACACTGCTCG-3′<br><br>5′- TGGYTKGCTTATTGGTTTGG-3′<br>5′-AATTCCCATCTCGCTATCACTTTC-3′	1656	95ºC for 3 min;<br>36 cycles; 95ºC for 30s, 64ºC for30s and 72ºC for 1 min<br>72ºC for 7 min<br>Annealing temperature 2nd round = 64.4ºC (ocelot samples) and 66ºC (jaguar samples)	Modified after Gou et al., 2012 [50]; Schreeg et al., 2016 [51]; Panait et al., 2021 [6]
**Piroplasmids (characterization)**	*cox-3*<br>-Cox3F<br>-Cox3R	5′-ACTGTCAGCTAAAACGTATC-3′<br>5′-ACAGGATTAGATACCCTGG-3′	600	94°C for 5 min;<br>50 cycles: 94°C for 20s, 55°C for 30s and 68°C for 45s;<br>72ºC for 7 min	[51,52]
**Piroplasmids (characterization)**	18S – 5.8S rRNA region<br>- Api18S rRNA-1690s<br>- Api5.8SrRNA-20as	5′- CTCCTACCGATCGAGTGATCCGGT -3′<br>5′- GCTGCGTCCTTCATCGTTGTGTGAG-3′	600	95ºc for 3 min;<br>45 cycles: 94ºC for 10s, 66ºC for 15s and 72ºC for 20s;<br>72ºC for 5 min	[53]

**Piroplasmids (characterization)**	5.8S – 28S rRNA region<br>- Api5.8SrRNA-20s<br>- Api28SrRNA-1as	5′-CTCACACAACGATGAAGGACGCAGC-3′<br>5′-CCGCTTARTTTTATGCTTAAATTCAGCGG-3′	400	95ºc for 3 min;<br>45 cycles: 94ºC for 10s, 66ºC for 15s and 72ºC for 20s;<br>72ºC for 5 min	Calchi et al. (2024)
