# Supplementary material for: Diversity of Cytauxzoon spp. (Piroplasmida: Theileriidae) in Wild Felids from Brazil and Argentina
Source: Pathogens. 2025 Feb 4;14(2):148. doi: 10.3390/pathogens14020148 (PMC12184619; doi:10.3390/pathogens14020148)
Supplement: Supplementary file 1 [file pathogens-14-00148-s001.zip › Supplementary material_3.pdf]

Table S3. BLASTn results of the sequences obtained in the amplification of five distinct molecular markers from *Cytauxzoon* spp. from wild felids blood samples.

Wild Felines ID (GenBank access number)	Locality	Gene	Sequence size (bp)	*Query-cover* (%)	*E-value*	Identity (%)	GenBank sequence (access number)	Genotype number
Panthera onca #172 (PQ686797)	Argentina	18S rRNA (~1500 bp fragment)	1381	100	0	99.86	*Cytauxzoon felis* detected in cat from USA (AY531524)	#3
Panthera onca #177 (PQ686792)	Espírito Santo- Brazil	18S rRNA (~1500 bp fragment)	1388	100	0	99.78	*Cytauxzoon felis* detected in cat from USA (AY531524)	#4
Panthera onca #180 (PQ686791)	Mato Grosso do Sul - Brazil	18S rRNA (~1500 bp fragment)	1381	100	0	99.86	*Cytauxzoon felis* detected in cat from USA (AY531524)	#3
Panthera onca #181 (PQ686787)	Goias - Brazil	18S rRNA (~1500 bp fragment)	1381	100	0	99.78	*Cytauxzoon felis* detected in cat from USA (AY531524)	#4
Panthera onca #229 (PQ686790)	Mato Grosso - Brazil	18S rRNA (~1500 bp fragment)	1379	100	0	99.78	*Cytauxzoon felis* detected in cat from USA (AY531524)	#4
Panthera onca #231 (PQ686789)	Mato Grosso do Sul - Brazil	18S rRNA (~1500 bp fragment)	1382	100	0	99.78	*Cytauxzoon felis* detected in cat from USA (AY531524)	#4
Panthera onca #234 (PQ686788)	Mato Grosso - Brazil	18S rRNA (~1500 bp fragment)	1379	100	0	99.78	*Cytauxzoon felis* detected in cat from USA (AY531524)	#4
Panthera onca #255 (PQ686798)	Argentina	18S rRNA (~1500 bp fragment)	1381	100	0	99.78	*Cytauxzoon felis* detected in cat from USA (AY531524)	#4
Panthera onca #292 (PQ686786)	Mato Grosso do Sul - Brazil	18S rRNA (~1500 bp fragment)	1380	100	0	99.86	*Cytauxzoon felis* detected in cat from USA (AY531524)	#3
Panthera onca #325 (PQ686793)	Mato Grosso do Sul - Brazil	18S rRNA (~1500 bp fragment)	1379	100	0	99.78	*Cytauxzoon felis* detected in cat from USA (AY531524)	#4

Panthera onca #351 (PQ686785)	Bahia - Brazil	18S rRNA (~1500 bp fragment)	1392	100	0	99.78	*Cytauxzoon felis* detected in cat from USA (AY531524)	#4
Panthera onca #355 (PQ686799)	Mato Grosso do Sul - Brazil	18S rRNA (~1500 bp fragment)	1381	100	0	99.78	*Cytauxzoon felis* detected in cat from USA (AY531524)	#4
Panthera onca #377 (PQ686794)	Para - Brazil	18S rRNA (~1500 bp fragment)	1380	100	0	99.78	*Cytauxzoon felis* detected in cat from USA (AY531524)	#4
Panthera onca #378 (PQ686795)	Para - Brazil	18S rRNA (~1500 bp fragment)	1380	100	0	99.86	*Cytauxzoon felis* detected in cat from USA (AY531524)	#3
Panthera onca #514 (PQ686796)	Paraná - Brazil	18S rRNA (~1500 bp fragment)	1389	100	0	99.78	*Cytauxzoon felis* detected in cat from USA (AY531524)	#4
Panthera onca #2 (PQ686800)	Goiás - Brazil	18S rRNA (~1500 bp fragment)	1465	100	0	99.86	*Cytauxzoon felis* detected in cat from USA (AF399930)	#3
Panthera onca #3 (PQ686801)	Goiás - Brazil	18S rRNA (~1500 bp fragment)	1466	100	0	99.80	*Cytauxzoon felis* detected in cat from USA (AF399930)	#4
Panthera onca #7 (PQ686802)	Goiás - Brazil	18S rRNA (~1500 bp fragment)	1466	100	0	99.80	*Cytauxzoon felis* detected in cat from USA (AF399930)	#4
Leopardus pardalis #8 clone 1 (PQ686803)	Mato Grosso do Sul - Brazil	18S rRNA (~1500 bp fragment)	1578	99	0	99.94	*Cytauxzoon brasiliensis* detected in ocelot from Brazil (GU903911)	#1
Leopardus pardalis #8 clone 3 (PQ686804)	Mato Grosso do Sul - Brazil	18S rRNA (~1500 bp fragment)	1578	99	0	99.87	*Cytauxzoon brasiliensis* detected in ocelot from Brazil (GU903911)	#1

Leopardus pardalis #8 clone 4 (PQ686805)	Mato Grosso do Sul - Brazil	18S rRNA (~1500 bp fragment)	1578	99	0	99.87	*Cytauxzoon brasiliensis* detected in ocelot from Brazil (GU903911)	#1
Leopardus pardalis #18 clone 1 (PQ686806)	Mato Grosso do Sul – Brazil	18S rRNA (~1500 bp fragment)	1545	100	0	99.87	*Cytauxzoon brasiliensis* detected in ocelot from Brazil (GU903911)	#2
Leopardus pardalis #18 clone 2 (PQ686807)	Mato Grosso do Sul – Brazil	18S rRNA (~1500 bp fragment)	1572	99	0	99.87	*Cytauxzoon brasiliensis* detected in ocelot from Brazil (GU903911)	#2
Leopardus pardalis #18 clone 3 (PQ686808)	Mato Grosso do Sul – Brazil	18S rRNA (~1500 bp fragment)	1543	99	0	99.87	*Cytauxzoon brasiliensis* detected in ocelot from Brazil (GU903911)	#2
Leopardus pardalis #18 clone 6 (PQ686813)	Mato Grosso do Sul - Brazil	18S rRNA (~1500 bp fragment)	1545	100	0	99.87	*Cytauxzoon brasiliensis* detected in ocelot from Brazil (GU903911)	#2
Leopardus pardalis #22 clone 1 (PQ686809)	Mato Grosso do Sul - Brazil	18S rRNA (~1500 bp fragment)	1578	99	0	99.94	*Cytauxzoon brasiliensis* detected in ocelot from Brazil (GU903911)	#1
Leopardus pardalis #22 clone 2 (PQ686811)	Mato Grosso do Sul – Brazil	18S rRNA (~1500 bp fragment)	1546	100	0	99.94	*Cytauxzoon brasiliensis* detected in ocelot from Brazil (GU903911)	#1
Leopardus pardalis #22 clone 6 (PQ686810)	Mato Grosso do Sul - Brazil	18S rRNA (~1500 bp fragment)	1548	100	0	99.94	*Cytauxzoon brasiliensis* detected in ocelot from Brazil (GU903911)	#1
Panthera onca #172 (PQ724028)	Argentina	*cytB*	1046	100	0	99.52	*Cytauxzoon felis* detected in cat from USA (KC207821)	#4
Panthera onca #177 (PQ724024)	Espírito Santo - Brazil	*cytB*	1076	100	0	99.54	*Cytauxzoon felis* detected in cat from USA (KC207821)	#4
Panthera onca #180 (PQ724023)	Mato Grosso do Sul - Brazil	*cytB*	1077	100	0	99,54	*Cytauxzoon felis* detected in cat from USA (KC207821)	#5

Panthera onca #181 (PQ724019)	Goiás - Brazil	*cytB*	1075	100	0	99.53	*Cytauxzoon felis* detected in cat from USA (KC207821)	#5
Panthera onca #229 (PQ724022)	Mato Grosso - Brazil	*cytB*	1077	100	0	99.54	*Cytauxzoon felis* detected in cat from USA (KC207821)	#5
Panthera onca #230 (PQ724031)	Maranhão - Brazil	*cytB*	1076	100	0	99.54	*Cytauxzoon felis* detected in cat from USA (KC207821)	#5
Panthera onca #231 (PQ724021)	Mato Grosso do Sul - Brazil	*cytB*	1077	100	0	99.54	*Cytauxzoon felis* detected in cat from USA (KC207821)	#5
Panthera onca #234 (PQ724020)	Mato Grosso - Brazil	*cytB*	1077	100	0	99.54	*Cytauxzoon felis* detected in cat from USA (KC207821)	#5
Panthera onca #255 (PQ724029)	Argentina	*cytB*	1075	100	0	99.53	*Cytauxzoon felis* detected in cat from USA (KC207821)	#5
Panthera onca #292 (PQ724018)	Mato Grosso do Sul – Brazil	*cytB*	1075	100	0	99.53	*Cytauxzoon felis* detected in cat from USA (KC207821)	#5
Panthera onca #325 (PQ724025)	Mato Grosso do Sul - Brazil	*cytB*	1077	100	0	99.54	*Cytauxzoon felis* detected in cat from USA (KC207821)	#5
Panthera onca #351 (PQ724017)	Bahia - Brazil	*cytB*	1077	100	0	99.54	*Cytauxzoon felis* detected in cat from USA (KC207821)	#5
Panthera onca # 355 (PQ724030)	Mato Grosso do Sul - Brazil	*cytB*	1076	100	0	99.54	*Cytauxzoon felis* detected in cat from USA (KC207821)	#5
Panthera onca #377 (PQ724037)	Para - Brazil	*cytB*	1087	100	0	99.54	*Cytauxzoon felis* detected in cat from USA (KC207821)	#5

Panthera onca #378 (PQ724026)	Para - Brazil	*cytB*	1079	100	0	99.54	*Cytauxzoon felis* detected in cat from USA (KC207821)	#4
Panthera onca #394 (PQ724032)	Minas Gerais - Brazil	*cytB*	1076	100	0	99.54	*Cytauxzoon felis* detected in cat from USA (KC207821)	#5
Panthera onca #514 (PQ724027)	Paraná - Brazil	*cytB*	1044	100	0	99.52	*Cytauxzoon felis* detected in cat from USA (KC207821)	#5
Panthera onca #7 (PQ724014)	Goiás - Brazil	*cytB*	1077	100	0	99.54	*Cytauxzoon felis* detected in cat from USA (KC207821)	#4
Panthera onca #9 (PQ724036)	Goiás - Brazil	*cytB*	1131	100	0	99.56	*Cytauxzoon felis* detected in cat from USA (KC207821)	#5
Panthera onca #20 (PQ724016)	Goiás - Brazil	*cytB*	1075	100	0	99.53	*Cytauxzoon felis* detected in cat from USA (KC207821)	#5
Leopardus pardalis #5L (PQ724033)	Mato Grosso do Sul - Brazil	*cytB*	1,087	94	0	99.03	*Cytauxzoon brasiliensis* detected in *Leopardus tigrinus* from Brazil (PP588457)	#8
Leopardus pardalis #14L (PQ724015)	Mato Grosso do Sul - Brazil	*cytB*	1,077	95	0	99.12	*Cytauxzoon brasiliensis* detected in *Leopardus tigrinus* from Brazil (PP588457)	#7
Leopardus pardalis #18L (PQ724034)	Mato Grosso do Sul - Brazil	*cytB*	1085	94	0	99.03	*Cytauxzoon brasiliensis* detected in *Leopardus tigrinus* from Brazil (PP588457)	#8
Leopardus pardalis #22L (PQ724035)	Mato Grosso do Sul - Brazil	*cytB*	1086	94	0	99.12	*Cytauxzoon brasiliensis* detected in *Leopardus tigrinus* from Brazil (PP588457)	#7

Panthera onca #180 (PQ724003)	Mato Grosso do Sul - Brazil	*cox-1*	1223	100	0	99.92	*Cytauxzoon felis* detected in *Felis silvestris* from USA (MT916252)	#2
Panthera onca #181 (PQ724001)	Goiás- Brazil	*cox-1*	1255	100	0	99.92	*Cytauxzoon felis* detected in *Felis silvestris* from USA (MT916252)	#2
Panthera onca #172 (PQ724012)	Argentina	*cox-1*	1221	100	0	99.92	*Cytauxzoon felis* detected in *Felis silvestris* from USA (MT916252)	#2
Panthera onca #177 (PQ724004)	Espírito Santo - Brazil	*cox-1*	1252	100	0	99.44	*Cytauxzoon felis* detected in *Felis silvestris* from USA (MT916252)	#4
Panthera onca #224 (PQ724006)	Amazonas - Brazil	*cox-1*	1254	100	0	99.92	*Cytauxzoon felis* detected in *Felis silvestris* from USA (MT916252)	#2
Panthera onca #231 (PQ724002)	Mato Grosso do Sul - Brazil	*cox-1*	1254	100	0	99.92	*Cytauxzoon felis* detected in *Felis silvestris* from USA (MT916252)	#2
Panthera onca #255 (PQ724013)	Argentina	*cox-1*	1040	100	0	99.42	*Cytauxzoon felis* detected in *Felis silvestris* from USA (MT916252)	-
Panthera onca #351 (PQ724000)	Bahia - Brazil	*cox-1*	1222	100	0	99.92	*Cytauxzoon felis* detected in *Felis silvestris* from USA (MT916252)	#2
Panthera onca #377 (PQ724005)	Pará - Brazil	*cox-1*	1254	100	0	99.92	*Cytauxzoon felis* detected in *Felis silvestris* from USA (MT916252)	#2

Panthera onca #394 (PQ724007)	Minas Gerais - Brazil	*cox-1*	1254	100	0	99.92	*Cytauxzoon felis* detected in *Felis silvestris* from USA (MT916252)	#2
Panthera onca #7 (PQ724011)	Goiás - Brazil	*cox-1*	1219	100	0	99.92	*Cytauxzoon felis* detected in *Felis silvestris* from USA (MT916252)	#4
Panthera onca #9 (PQ724008)	Goiás - Brazil	*cox-1*	1222	100	0	99.92	*Cytauxzoon felis* detected in *Felis silvestris* from USA (MT916252)	#2
Panthera onca #12 (PQ724010)	Goiás - Brazil	*cox-1*	1222	100	0	99.43	*Cytauxzoon felis* detected in *Felis silvestris* from USA (MT916252)	#2
Leopardus pardalis #6L (PQ724009)	Mato Grosso do Sul - Brazil	*cox-1*	1254	99	0	91.46	*Cytauxzoon felis* detected in *Felis silvestris* from USA (MT916250)	#6
Leopardus pardalis #5L (PQ687019)	Mato Grosso do Sul - Brazil	ITS-1	450	96	0	100	*Cytauxzoon* sp. detected in cat from Brazil (KP683154)	-
Leopardus pardalis #21L (PQ687020)	Mato Grosso do Sul - Brazil	ITS-1	450	96	0	100	*Cytauxzoon* sp. detected in cat from Brazil (KP683154)	-
Leopardus pardalis #5L (PQ687021)	Mato Grosso do Sul - Brazil	ITS-2	237	100	$2X10^{-101}$	95.82	*Cytauxzoon* sp. detected in *L. pardalis* from Brazil (FJ876458)	-
Leopardus pardalis #21L (PQ687022)	Mato Grosso do Sul - Brazil	ITS-2	237	100	$5X10^{-98}$	94.98	*Cytauxzoon* sp. detected in *L. pardalis* from Brazil (FJ876458)	-
